# Editorial: Immune modulation in tumor microenvironment: New perspectives for cancer immunotherapy

**DOI:** 10.3389/fcell.2022.1103705

**Published:** 2023-01-04

**Authors:** Zimu Deng, Xuejun Sun, Jian Cao, Qian Xiao

**Affiliations:** ^1^ Shanghai Institute of Biochemistry and Cell Biology, Chinese Academy of Sciences (CAS), Shanghai, China; ^2^ Xi’an Jiaotong University, Xi’an, China; ^3^ Rutgers Cancer Institute of New Jersey, Rutgers, The State University of New Jersey, New Brunswick, NJ, United States

**Keywords:** tumor microenvironment, immunotherapy, immune suppression, inflammation, cancer progression

## Introduction

The advances in immunotherapies in the last decades have brought new hope in the battle against cancer (Wang et al., 2019; Morad et al., 2021). These therapies include immune checkpoint blockade (ICB), chimeric antigen receptors T-cell (CAR-T), T-cell receptor (TCR)-based adoptive cell therapy, oncolytic virus, and cancer vaccine. Unfortunately, intrinsic and acquired resistance prevent most patients from benefiting from these emerging treatments (Majzner and Mackall, 2018; Morad et al., 2021). Tumor cells are heterogeneous and able to harness epigenomic alterations to alter the phenotype. Besides cancer cells, endothelial cells, immune cells, tumor-associated fibroblasts, extracellular matrix components, metabolic products, and signaling molecules form structurally complicated tissues known as the tumor microenvironment (TME) (Hu et al., 2022). The heterogeneity of TME provides unique opportunities for tumors to escape immune restriction through various mechanisms (Zhengxi Chen, 2020; Hu et al., 2022). Therefore, discovering and targeting these immune escape mechanisms have been a focused area in cancer immunology, aiming to develop new strategies to improve immunotherapies (Bader et al., 2020; Hu et al., 2020; Hu et al., 2022).

This Research Topic focuses on the immunosuppressive TME in tumor initiation, progression, and resistance to immunotherapies. 16 outstanding original studies and 2 review articles from 139 authors have been published, demonstrating emerging interest in cancer immunotherapy. This Research Topic contains studies on the following three topics.

## Immune modulators in TME

**FIGURE 1:**
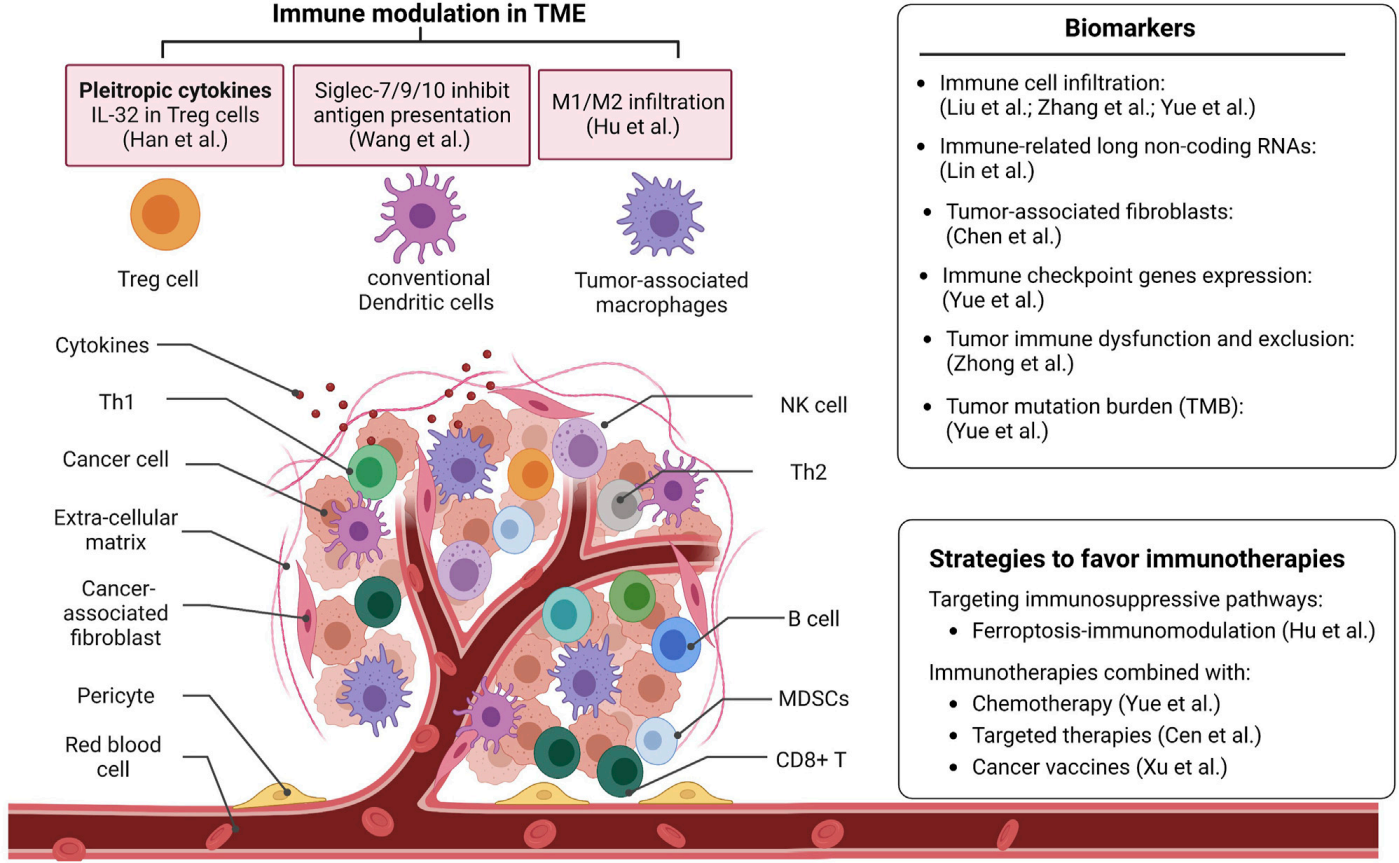
The representative studies from the Research Topic advance the understanding of the tumor microenvironment. This Research Topic also provides new biomarkers for predicting immune checkpoint blockade therapy and approaches to favor immunotherapies. NK cell: Natural Killer cell; Th1: Type 1T helper; Th2: Type 2 T helper; MDSCs: Myeloid-derived suppressor cells.

Tumors are heterogeneous. It is a complex structure tissue comprised of various types of cells (Figure 1). Those immunosuppressive or immune escape mechanisms are initiated by tumor cells or non-tumor cells to suppress the antitumor immune response. A comprehensive understanding of the TME favors effective immune therapy (Binnewies et al., 2018).

Abnormal cytokine secretion in TME is important in suppressing tumor immune response. In this Research Topic, Han et al. analyzed the single-cell RNA sequencing data to investigate the function of Interleukin-32 (IL-32) in the TME of esophageal squamous cell carcinoma (ESCC). They found that IL-32 is highly expressed in CD4^+^ regulatory T cells (Treg cells). Knockdown of IL-32 reduced Foxp3 expressions in CD4^+^ T cells, which indicates IL-32 may be used as a target for ESCC cancer immunotherapy.

Cell-cell interaction proteins, such as Siglecs (Sialic acid-binding immunoglobulin-like lectins), have also been reported as potential new ICB molecules. Wang et al. found that classical conventional dendritic cells (DCs) highly expressed inhibitory Siglecs, such as Siglec-7, Siglec-9, and Siglec-10, in human cancer samples. Consistently, they found that the expression of the Siglec-E receptor is upregulated on tumor-associated cDCs *in vivo* murine tumor models. Expressing these inhibitory Siglecs in DC cell lines and bone marrow-derived DCs showed impaired maturation states. Furthermore, depletion or inhibition of these inhibitory Siglecs on DCs enhanced the priming process of antigen-specific T cells and induced T cell proliferation. This study enhanced the understanding of the inhibitory functions of Siglecs on DCs and revealed new potential targets for cancer immunotherapy.

## Biomarkers to predict immune checkpoint blockade (ICB) therapy

Identifying novel biomarkers to predict the response of cancer patients to ICB therapy is a challenge for immunotherapy (Crow et al., 2019). Immune cell infiltration is one of the most important signatures or indicators. A study from this Research Topic shows that myeloid dendritic cells and B cells are prognostic factors independently, which could predict ICB efficacy for lung adenocarcinoma and lung squamous cell carcinoma patients, respectively. Another study in colorectal cancers revealed two robust immune subtypes: the “immune cold subtype”, characterized by the deficiency and depletion of immune cells; and the “immune hot subtype”, characterized by the abundance of immune cell infiltration and ECM protein activation. Furthermore, they found that loss of MHC molecules and insufficient tumor antigen presentation are immune escape mechanisms in the “immune cold subtype” tumors. This study provided a deep understanding of TME in colorectal cancers. The abundance of macrophage (DeNardo and Ruffell, 2019) and some specific gene (PD-L1 and CD8A) signatures were identified and could be used to predict immunotherapy efficacy. Integrins, including Integrin alpha L, expressed in immune cells, were associated with cancer patient prognosis and potentially be applied to cancer therapy as biomarkers and targets (Hayat et al., 2020).

Besides immune cell infiltration signatures, other signatures are also being used as biomarkers. In this Research Topic, Lin et al. characterized that immune-related long non-coding RNAs (irlncRNAs) are correlated with immune cell infiltration signature and chemosensitivity in patients with soft tissue sarcoma. In addition, tumor-associated fibroblasts regulate the recruitment and function of immune cells *via* secreting cytokines/chemokines or remodeling the matrix, creating an immunosuppressive TME (Barrett and Pure, 2020). Here, Chen et al. found that the percentage of tumor-associated fibroblasts in the tumor tissue was associated with tumor immune characteristics and clinical outcomes of gliomas. They established a prediction model based on tumor-associated fibroblasts related gene signatures to predict the response of patients to immunotherapy.

Another important signature is the metabolism of cancer cells. The TME imposes massive metabolic restrictions on antitumor NK and T cells (DePeaux and Delgoffe, 2021). Heme oxygenase 1, an essential enzyme in heme catabolism, and HMOX1-related genes (HRGs) were found to regulate the immune-related pathways. HMOX1 expression could be used as a predictor for the response of immunotherapies in patients with Lower-grade glioma. Strategies or methods that target metabolic restrictions would break metabolic barriers of therapy.

TME signatures include immune cell infiltration score, tumor immune dysfunction and exclusion score, stromal score, tumor mutation burden value, and immune checkpoint genes expression score. Those emerging TME signatures have been identified in multiple tumor types with low or high immunogenicity. However, a systematic investigation of the TME needs to be validated to help clinicians predict the outcome of immunotherapy, facilitate clinical decision-making, and develop personalized treatment.

## Strategies/approaches to favor immunotherapies

Tumor cells suppress the immune environment through different mechanisms (Zhou et al., 2021). Recently, diversified therapeutic strategies have been used to restore host immunity and enhance the sensitivity to immunotherapy (Binnewies et al., 2018; Hu et al., 2022). Ferroptosis in tumors, an iron-dependent non-apoptotic cell death, has a dual role in tumor promotion and suppression. The driver gene SOCS1 and suppressor gene FTH1 of ferroptosis are correlated with the infiltration of M1/M2 macrophage in the head and neck squamous cell carcinoma, respectively, which indicates ferroptosis-immunomodulation may be targeted and provide a new strategy for enhancing the efficacy of immunotherapy.

A major barrier to antitumor immunotherapies is acquired resistance (Schoenfeld and Hellmann, 2020). Numerous efforts have been made to find novel approaches to enhance immunotherapy, such as combination treatment of checkpoint inhibitors with chemotherapy or target therapy (Larkin et al., 2015; Hu et al., 2020). Chemotherapy such as cisplatin enhances antitumor T cell responses, leading to a better therapeutic effect when combined with ICB therapy. A study on this Research Topic showed that two cisplatin resistance-related genes, CCL18 and BCL2A1, are novel biomarkers for combined therapy of cisplatin and ICB in colorectal cancer patients. Targeted therapies inhibit oncogenic proteins and their regulated signaling pathways. Recently, more and more studies combined targeted therapies and immunotherapies to unleash patient antitumor immunity. A study from this Research Topic found more neutrophils and macrophages M1 infiltrate in BRAF-mutated colon tumors compared to BRAF-wt colon tumors. The immunotherapeutic molecules, including CTLA-4, PD-1, PD-L1, LAG-3, and TIM-3, were upregulated in BRAF-mutated tumors, which shed light on the combination therapy of ICB and BRAF inhibitors in this subgroup of patients.

Furthermore, cancer vaccines, especially mRNA vaccines, are emerging as a feasible strategy for cancer therapy (Sahin and Tureci, 2018; Miao et al., 2021). Xu et al. screened for genes positively correlated with antigen-presenting cell infiltration in lung adenocarcinoma. CBFA2T3 and KLRG1 are identified as potential tumor antigens used in mRNA vaccines in lung adenocarcinoma. They also identified the biomarkers to assess immunogenicity for mRNA vaccines. Overall, we expect a new perspective for combining mRNA vaccine and immunotherapy in future personalized cancer treatments.

## Conclusion

This Research Topic presents recent discoveries on the immunomodulation of TME and its effects on immunotherapies (Figure 1). With these studies, this Research Topic aims to provide new prognostic biomarkers and novel insights into combination therapy strategies for cancer treatment.
